# Identifying nutritional, functional, and quality of life correlates with male hypogonadism in advanced cancer patients

**DOI:** 10.3332/ecancer.2015.561

**Published:** 2015-08-04

**Authors:** Domenico Fuoco, Jonathan di Tomasso, Caroline Boulos, Robert D Kilgour, Jose A Morais, Manuel Borod, Antonio Vigano

**Affiliations:** 1 McGill Nutrition and Performance Laboratory, McGill University Health Centre, Montreal H4A 3S5, Canada; 2 Department of Exercise Science, Concordia University, Montreal H4B 1R6, Canada; 3 Department of Geriatric Medicine, McGill University, Montreal H4A 3S5, Canada; 4 Division of Supportive and Palliative Care Medicine, McGill University Health Centre, Montreal H4A 3S5, Canada

**Keywords:** hypogonadism, male, quality of life, function, testosterone, cachexia, cancer, case series study

## Abstract

With the availability of a potential treatment to reverse male hypogonadism (MH), the primary aim of this case series study was to determine independent relationships between this condition and the nutritional, functional, and quality of life characteristics of advanced cancer patients (ACP). Free testosterone levels were measured in 100 male patients with advanced lung and gastrointestinal (GI) cancer. Routine blood markers of nutrition and inflammation, self-reporting questionnaires for symptom, nutrition, and functional status along with handgrip dynamometry were assessed for all patients at bedside. Almost half of this cohort underwent further assessments (body composition, lower body strength, in depth quality of life and fatigue questionnaires) at the McGill Nutrition and Performance Laboratory (mnupal.mcgill.ca). Multiple regression analyses were performed to identify independent correlations between free testosterone and the above measures. Seventy-six percent of patients were diagnosed with MH. Using multiple linear regression, low free testosterone (31.2 pmol/L) was independently associated with lower albumin (B = –3.8 g/L; 95% confidence interval CI –6.8:–0.8), muscle strength (–11.7 lbs; –20.4: –3.0) and mass in upper limbs (–0.8 kg; –1.4: –0.1), overall performance status (Eastern Cooperative Oncology Group Performance Scale, ECOG PS 0.6; 0.1:1.1), cancer-related fatigue (Brief Fatigue Inventory, BFI 16.7; 2.0: 31.3), and overall quality of life (MQoL total score –1.42; –2.5: –0.3). Thus MH seems to be highly prevalent in ACP, and it is independently associated with important nutritional, functional, and quality of life characteristics in this patient population.

## Introduction

MH is characterised by the presence of low circulating concentrations of androgens, namely testosterone, a hormone mainly produced by the testicles [1]. Androgen deficiency has been found to be highly prevalent in male cancers and it has been suggested as a contributing factor of cachexia sequelae and cancer-related fatigue [2]. In addition, MH could be an important cause of muscle wasting in cancer cachexia and sarcopoenia [2]. Clinical signs such as high levels of inflammation, low appendicular fat and muscle mass, and questionnaire scores indicative of anxiety, depression, and poor functional well-being, have been found to be associated with MH primarily via univariate analyses [2]. Recently, Dev *et al* (2014) confirmed the association between MH and survival through multivariate analyses [3]. The aim of this case series study is to determine, using multivariate analyses, whether independent relationships exist between MH and nutritional, functional, and quality of life characteristics in ACP.

## Methods

### Population

The work herein discusses a clinical study classified according to the National Cancer Institute (NCI) in consecutive case series. All patients recruited for this study were male and had a new diagnosis (six months or less) of advanced (stage III–IV) GI or non-small cell lung (NSCL) cancers within the Cancer Mission of the McGill University Health Centre. The patient recruitment and data collection took place between March 2006 and November 2007. Ethical approval was acquired from the Institutional Review Board of the McGill University Health Centre.

### Measures

The diagnosis of MH was made on the basis of serum-free testosterone measurement, using the Coat-A-Count (Diagnostic Product Corp., Los Angeles, CA) radioimmunoassay, and ADAM questionnaire [12]. Concentrations lower than 31.2 pmol/L were considered to be clinically significant for MH, as already reported by Gagnon *et al* [7].

All other assessments took place either in the hospital setting (at bedside or in outpatient clinics) or at the McGill Nutrition and Performance Laboratory (MNUPAL, www.mnupal.mcgill.ca). MNUPAL is a clinical outcome research laboratory where patients affected by multiple chronic diseases such as cancer in advanced stages can receive the best supportive care and medical oversight in a new state-of-the-art facility. In the hospital setting, the following tests were completed: the Edmonton Symptom Assessment System (ESAS) to assess severity of anorexia, weakness, dyspnoea, and decreased feeling of well-being; the abridged Patient Generated Subjective-Global Assessment (aPG-SGA) to evaluate nutritional status; and handgrip strength by Jamar dynamometry (Sammons Preston, Bolingbrook, IL). Selected blood markers were also measured to assess the presence/severity of the inflammatory response (white blood cells count, C-reactive protein) and visceral protein status (serum albumin).

At MNUPAL, the patients completed the BFI [22] and the McGill Quality of Life questionnaire (MQoL) [23]. Body composition was assessed via dual-energy x-ray absorptiometry (DXA) (Lunar Prodigy, GE Healthcare, Madison, WI). Scans were analysed for total lean and fat mass (kg), percentage of fat and appendicular lean tissues, along with bone mineral content and bone mineral density, using the Advance Encore software (GE Healthcare).

All data was stored in the MNUPAL Human Cancer Cachexia Database (HCCD).

### Statistical analysis

In order to test for independent relationships between low free testosterone levels (independent variable) with the above clinical and biological outcomes (dependent variables), we performed several multivariate linear regression models (one for each dependent variable), controlling for gender, age, time between diagnosis, and baseline assessment, survival time from baseline assessments to death/loss to follow-up, diagnosis of lung versus GI malignancies, presence/absence of concurrent oncological treatment (radio/chemotherapy), and concurrent medications. For medications potentially impacting on the relationships under investigation, the presence or absence of at least one of the following medications was considered: statins, anti-inflammatories, steroids, angiotensin-converting-enzyme (ACE) inhibitors, anti-hormonal agents, antioxidants, essential amino acids, anabolic hormones, and metformin. All data were analysed using SPSS (version 14.0, 2005, SPSS Inc., Chicago, IL).

## Results and discussion

### Results

At the time of the final analysis, 100 patients satisfied the inclusion criteria and were recruited for this study. All patients were assessed at the hospital bedside, whereas 48 of them (48%) were also assessed at the MNUPAL. Table 1 indicates the clinical characteristics (n = 100). Patients had an average age of 64 years old (SD ± 11.5) and presented predominantly with GI malignancies (62%). The presence of MH was identified in 76 patients (76%). A statistically significant indirect association was found between MH and albumin levels (p = 0.01). MH was also associated with higher levels of fatigue, poor performance status, and quality of life. Significantly lower handgrip strength correlated with MH as well as decreased muscle mass in the upper limbs. However, there was no statistically significant relationship between MH and symptom severity or biological markers of inflammation (p > 0.2).

### Discussion

The study shows that in a sample of 100 patients, there is a high prevalence of hypogonadism in males with advanced cancer. In addition, there is an independent relationship between MH and a worsening of visceral protein status, muscle strength, and mass in upper limbs, overall performance status, cancer-related fatigue, functional, and overall quality of life.

According to Burney and Garcia (2012), MH has been reported between 40% and 90% in ACP [4]. These findings concur with the high prevalence (76%) of MH in the present study. The complex pathophysiology of androgen deficiency in ACP has not been entirely elucidated. The presence of higher levels of circulating inflammatory mediators and/or concurrent oncological treatments have been shown in a combination of primary (i.e. related to testicular failure) and secondary (i.e. related to the hypothalamus–pituitary axis failure) hypogonadism [4]. The decline in testosterone levels observed in ACP may be negatively influenced by various factors such as other comorbidities [13], chronic opioid treatments [14], and changes in concentrations of sex-hormone-binding globulin [2].

Since the late 1980’s, physicians have agreed that MH is a non-specific consequence and a biomarker of chronic illness [13]. The current hypothesis to explain MH is based on the body’s adaptive response to the illness. During the recovery phase from illness, changes in homeostasis reflect a tendency to conserve energy, reduce aggressiveness, depresses libido and sexual activity. [13,15–17].

ACP present with symptoms of fatigue and cancer-related pain with the latter being typically treated with long-term opioid therapy [14]. Although cancer-related fatigue and pain were common symptoms in this clinical study, opioid therapy was not monitored. We have considered these facts as limitations of our study. Also because MH is a consequence of chemotherapy [18] and of the cancer per se [21], we did not report the percentage of those treated with or without opioids.

Handgrip strength as well as performance status (as measured by box 4 in the aPG-SGA) were clearly lower in the presence of androgen deficiency and this observation concurred with the presence of decreased lean mass in the arms and fatigue in hypogonadic patients. Kilgour *et al* (2010) have recently identified an association between muscle strength and muscle mass in ACP such that those who experienced greater fatigue had lower muscle mass and strength [5]. All of the above associations suggest that the lack of circulating testosterone could have a blunted anabolic impact on muscle morphology in cancer patients with MH. This hypothesis has been supported by eight clinical, randomised, double-blinded, and placebo-controlled trials using testosterone but in a rather small cohort population of hypogonadic patients who were sarcopoenic with no diagnosis of cancer (https://clinicaltrials.gov/ct2/results?term=testosterone&cond=%22Sarcopenia%;22). To date, only one clinical trial with testosterone administration has been conducted in ACP (https://clinicaltrials.gov/ct2/show/NCT00472940?term=testosterone&cond=%22Cachexia%22+AND+%22Cachexia%22&rank=7). The results of this trial are sufficient to improve fatigue scores but not Functional Assessemnt of Chronic Ilness Therapy Fatigue (FACIT-F) quality of life measures [19]. An earlier retrospective study demonstrated a clear association between MH and a decrease in quality of life with poor survival [3]. These data are in agreement with the pivotal work of Traish *et al* (2011), who showed that a reversal of MH in ACP improves quality of life and survival [20].

In a Phase II clinical trial using Enobosarm, a selective androgen receptor modulator (SARM), Dobs *et al* (2011) reported an increase lean body mass when compared to the placebo [6]. In the same study, the patients on SARM were found to have a greater functional ability as measured by the stair climbing test [6] suggesting that the drug-induced increase in muscle mass may be related to the improvement in functional ability.

As mentioned above, we found an independent association between levels of free testosterone and albumin but no relationship between MH and C-reactive protein and white blood cell counts. The latter relationship is in contrast with other studies where androgen deficiency was clearly associated with high C-reactive protein levels [3, 8, 9]. There could be several factors to explain this discordance, including different clinical characteristics among the sample populations such as different cancer primaries, stages, and co-morbidities. Another possible explanation for lack of any relationship between MH and inflammation between our study and others could be related to the type of statistical analysis (e.g., univariate versus multivariate analyses) [7].

The multiple regression models showed independent correlations of MH with cancer-related fatigue (CRF), a trend towards self-reported weakness (p = 0.09) and quality of life in our patient population. However, multivariate analysis did not confirm the associations between the sense of well-being, dyspnoea, and anorexia with MH, which were found by Dev *et al* (2014) via univariate analysis. In this work, we conducted multivariate analyses taking into account the statistical influence of the covariates. There is a potential weakness in using univariate analysis when an independent variable (i.e., MH) may have more than one dependent variable influencing the statistical outcome. It is possible that the covariates used in our multiple regression analysis altered the relationship between MH and other potential correlates so much so that the significant univariate findings were lost. Fatigue has been frequently linked with androgen deficiency in malignant chronic conditions [10, 11]. This strong relationship between cancer-related fatigue (CRF) and hypogonadism indicates the need to rule out the presence of the latter condition whenever clinicians are looking for an improvement of CRF in ACP.

Our study is limited in its retrospective design and sample size. Furthermore, we only assessed androgen status via free testosterone levels, without measuring sex hormone binding globulin or luteinising hormone (LH) to better determine the causes of MH. The purpose of this paper was not to determine the cause of MH in advanced cancer, as if then would have required a vastly different design and sample size. In a recent review, Miner *et al* (2014) reported over 24 different aetiologies of MH, with ten primary, seven secondary, and seven mixed (primary and secondary) hypogonadism. However, the main goal of this study was not to establish the exact aetiology of hypogonadism (primary or secondary), but to better understand how the different factors are clinically relevant to MH in ACP.

## Conclusions and future perspectives

In conclusion, MH appears to be highly prevalent and clinically significant in advanced cancer patients. Routine screening and treatment of this condition could allow for significant improvement in overall nutrition, function, and quality of life. The main concept emerging from this case series analysis, even if limited in the sample, is the importance to assess any degree of MH. In fact, other authors are investigating the role of MH in wasting diseases, such as sarcopoenia and cachexia. In the current clinical practice, to better assess the symptoms of burden, depression, and fatigue scales of ACP, serum testosterone values should be included. Several questions remain to be answered. For example, does MH need to be present for muscle wasting to occur? Will the treatment of MH restore or help to maintain muscle mass, make patients stronger so that they can be more mobile, reduce fatigue levels, have a better QoL, and improve the other clinical correlates (e.g., improve albumin levels and reduce inflammation)?

This paper is part of an investigation that our laboratory is conducting on ACP. The next step will be to design a large population-based clinical trial in collaboration with other cancer centres to determine the importance of MH in the evolution of muscle wasting diseases.

## Author Contributions

Antonio Vigano conceived the project and had a primary role and responsibility for data collection and analysis, manuscript preparation, and submission.

Caroline Boulos participated in the preparation of the first version of the manuscript.

Robert Kilgour and Jose Morais provided their expertise for data analysis and manuscript preparation.

Domenico Fuoco, Jonathan Di Tomasso, and Manuel Borod participated in the preparation of the final manuscript and its submission.

## Conflicts of Interest

The authors have no financial or other relations that could lead to conflict of interest.

## Ethics

All subjects gave their written informed consent for inclusion before they participated in the study. The study was conducted in accordance with the Declaration of Helsinki. Ethical Approval was acquired from the Institutional Review Board of the McGill University Health Centre. The responsibility of the study was assigned to Dr Antonio Vigano as principal investigator and coordinator of the project. The project identification code is MN9614AV, and it was attributed to the McGill Nutrition and Performance Laboratory as the funding entity of the project.

## Acknowledgments

The authors thank Dr Peter Chan and Dr Lodovico Balducci for having reviewed this manuscript. Dr Chan is medical director of the Male Reproductive Medicine Department and urological surgeon at McGill University Health Centre, Montreal, Canada. Dr Balducci is a geriatric oncologist at the Moffit, Tampa (Florida, USA), he received the inaugural BJ Kennedy Award for Scientific Excellence in Geriatric Oncology in 2007 from the American Society of Clinical Oncology (ASCO).

## Figures and Tables

**Table 1.** Patient characteristics, demographics, and ongoing treatments present at the assessment visit.

Age (years) (mean ± SD)	64.0 ± 11.5	
Weight (kg) (mean ± SD)	75.0 ± 17.0	
Body mass (kg/m^2^) (mean ± SD)	25.0 ± 5.3	
Tumour type (%)	NSCL	38.0
	GI	62.0
Number of hypogonadic patients (%)	76 (76%)	
Radiotherapy (%)	yes	19.0
	no	81.0
Chemotherapy (%)	yes	39.0
	no	61.0

**Table 2.** Multiple linear regression analysis comparing hypogonadic (free testosterone < 31.2 pmol/L) versus eugonadic patients.

Variables	B	95% CI	p-value	n
White blood cells (10^9^/L)	0.6	–1.2; 2.40	>0.20	100
C-reactive protein (mg/L)	–0.6	–25.3; 24.2	>0.20	≈
Albumin (g/L)	–3.8	–6.8; –0.8	0.01	≈
BMI (kg/m^2^)	–1.8	–4.5; 0.7	0.15	≈
ESAS well-being (0–10 worse)	0.3	–0.9; 1.6	>0.20	≈
ESAS weakness (0–10 worse)	1.3	–0.2; 2.8	0.09	≈
ESAS dyspnoea (0–10 worse)	–0.1	–1.5; 1.2	>0.20	≈
ESAS anorexia (0–10 worse)	1.0	–0.6; 2.5	>0.20	≈
aPG-SGA Weight loss one month (%)	–0.06	–2.7; 2.6	>0.20	≈
aPG-SGA Weight loss six months (%)	2.0	–2.5; 6.5	>0.20	≈
aPG-SGA box 4 (0–4 worse)	0.6	0.1; 1.1	0.03	≈
aPG-SGA boxes 1–4 (0–36 worse)	2.8	–0.3; 5.9	0.08	≈
BFI Total (0–100 worse)	16.7	2.0; 31.3	0.03	≈
MQoL Physical score (10–0 worse)	–2.2	–4.0; –0.4	0.02	≈
MQoL Total score (10–0 worse)	–1.42	–2.5; –0.3	0.01	≈
Handgrip average (lbs)	–11.7	–20.4; –3.0	<0.01	≈
Handgrip percentile	–11.2	–20.6; –1.9	0.02	≈
Total fat mass* (kg)	–5.5	–14.0; 3.0	0.20	48
Percent fat* (%)	–4.3	–12.3; 3.6	>0.20	≈
Total lean mass* (kg)	–2.4	–7.5; 2.72	>0.20	≈
Percent lean* (%)	4.6	–2.7;12.0	>0.20	≈
Arms lean mass* (kg)	–0.8	–1.4; –0.1	0.03	≈
Legs lean mass* (kg)	–1.1	–3.1; 1.0	>0.20	≈
Bone mineral content* (g)	–99.3	–452.0; 253.4	>0.20	≈
Bone mineral density* (g/cm^2^)	–0.02	–0.1; 0.06	>0.20	≈

BMI = Body Mass Index, ESAS = Edmonton Symptom Assessment System, aPG-SGA = abridged Patient Generated Subjective Global Assessment, MQoL = McGill Quality of Life.

* Measured by dual energy x-ray absorptiometry (DXA)
